# A New Efficient Hybrid Intelligent Model for Biodegradation Process of DMP with Fuzzy Wavelet Neural Networks

**DOI:** 10.1038/srep41239

**Published:** 2017-01-25

**Authors:** Mingzhi Huang, Tao Zhang, Jujun Ruan, Xiaohong Chen

**Affiliations:** 1 Department of Water Resources and Environment, Guangdong Provincial Key Laboratory of Urbanization and Geo-simulation, Sun Yat-sen University, Guangzhou 510275, PR China; 2 School of Environmental Science and Engineering, Guangdong Provincial Key Laboratory of Environmental Pollution Control and Remediation Technology, Sun Yat-Sen University, Guangzhou 510275, PR China

## Abstract

A new efficient hybrid intelligent approach based on fuzzy wavelet neural network (FWNN) was proposed for effectively modeling and simulating biodegradation process of Dimethyl phthalate (DMP) in an anaerobic/anoxic/oxic (AAO) wastewater treatment process. With the self learning and memory abilities of neural networks (NN), handling uncertainty capacity of fuzzy logic (FL), analyzing local details superiority of wavelet transform (WT) and global search of genetic algorithm (GA), the proposed hybrid intelligent model can extract the dynamic behavior and complex interrelationships from various water quality variables. For finding the optimal values for parameters of the proposed FWNN, a hybrid learning algorithm integrating an improved genetic optimization and gradient descent algorithm is employed. The results show, compared with NN model (optimized by GA) and kinetic model, the proposed FWNN model have the quicker convergence speed, the higher prediction performance, and smaller RMSE (0.080), MSE (0.0064), MAPE (1.8158) and higher R^2^ (0.9851) values. which illustrates FWNN model simulates effluent DMP more accurately than the mechanism model.

In recent years, phthalic acid esters (PAEs) have caught extensive concerns because they are widely used as plastic plasticizers, and additives in more than a hundred varieties of products, such as toy, packing material cosmetics production. Therefore, these persistent and toxic organic compounds, which could harm the health of organisms and human by transmission of food chain and bioaccumulation, commonly exists in various environments. Dimethyl phthalate (DMP) known as one of the most important and extensively used PAEs, has been already measured in various environment, such as various surface water, groundwater, sediments of water, atmosphere, aerosol particle, soil^1^,^2^,^3^,^4^, and is known to likely cause dysfunctions of the endocrine systems, liver, and nervous systems of humans and animals^5^,^6^. Therefore, DMP has been listed as a priority control pollutant by American Environmental Protection Agency (USEPA)^7^, Ministry of Environmental Protection of the People’s Republic of China^8^ and European Union (EU)^9^.

In the past few years, previous studies have demonstrated that several PAEs can take the biodegradation under aerobic conditions and anaerobic conditions^10^,^11^,^12^,^13^,^14^,^15^, in activated sludge^16^,^17^ and in acclimated sludge^18^,^19^,^20^,^21^. In order to clearly investigate the degradation mechanism and behavior of DMP in the treatment system, various mathematical models are proposed to describe the degradation and behavior of DMP. However due to the highly nonlinearity and complexity of degradation mechanism for DMP, traditional mathematical methods are hard to exactly to model and simulate the biodegradation process^22^.

In recent years, artificial Intelligence (AI), which can overcome the restrictions of the traditional modeling methods and efficiently approximate any nonlinear processes, have been utilized for simulation, prediction and modeling^23^,^24^,^25^. Among AI methodologies and approaches, neural network (NN) is the most known and popular and has been widely used on account of its universal approximation properties^26^. Although NN can be used for forecasting the effluent quality parameters from wastewater treatment process (WWTP), there are also some shortcomings for NN, such as easily getting into local minima, low learning efficiency, slow convergence rate and difficultly extracting the mapping rules and so on^27^,^28^.

To solve the drawbacks of NN, a great number of new hybrid intelligent techniques have been constructed, such as fuzzy neural network and wavelet neural network (WNN). WNNs, which take the advantages of NN and WT, are designed by using wavelet functions as the neuron’s activation functions and can be regarded as the function-linked networks based wavelet function. Due to the good time-frequency localization characteristics of wavelets, wavelet function is an important tool in functional approximation. Therefore, the learning and memory ability of WNN is more efficient than conventional NN in the light of network size, convergence rate and accuracy^29^,^30^. Nevertheless, there is also a shortcoming for WNN^31^, which is difficult to understand the mapping rules. This is exactly the advantage of fuzzy logic (FL).

Therefore, combining the advantages of NN, FL and WT, a novel hybrid intelligent technique- fuzzy wavelet neural network (FWNN), which make effective use of self learning and memory abilities of NN, handling uncertainty capacity of FL and analyzing local details superiority of WT, could be constructed to enhance the abilities of approximation accuracy, convergence rate and generalization^26^. So compared with other conventional modeling techniques, the hybrid FWNN provide a more powerful way for process modeling, simulation and optimizing, particularly for complex wastewater treatment process.

In this work, a novel FWNN, which uses the concepts of FL in combination with WNN, was proposed for modeling and simulating biodegradation process of DMP in an AAO wastewater treatment process. The degradation and behavior of DMP were investigated, a degradation model including biodegradation and sorption using the proposed FWNN model was formulated so as to evaluate the fate of DMP. In order to avoid the trial-and-error process and the impact coming from random initialization, a hybrid learning algorithm integrating an improved genetic algorithm (GA) and gradient descent algorithm (GDA) was adopted.

## Materials and Methods

### Reactor system

**Figure 1:**
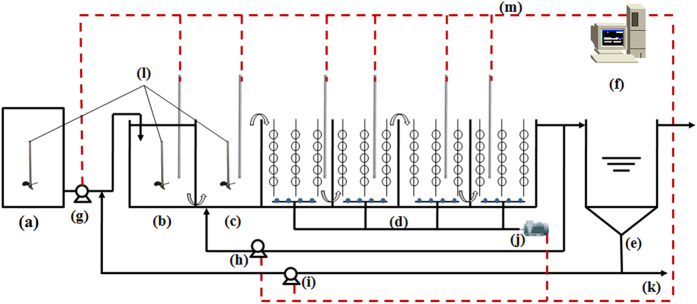
Schematic diagram of AAO system. (a) regulating tank, (b) anaerobic zone, (c) anoxic zone, (d) aerobic zone, (e) settler, (f) computer monitoring system, (g) inlet pump, (h) reflux pump for mixed liquor, (i) return sludge pump, (j) air blower, (k) the wasted sludge, (l) mixer, (m) the signal collecting for DO, ORP, pH, and Q.

As shown in Fig. 1, the AAO treatment system made of polyethylene includes mainly four parts: one anaerobic zone with volume of 40 litres, one anoxic zone with volume of 40 litres, three aerobic zone with 160 litres and one settling zone. There were two motor-driven stirrers employed in anaerobic and anoxic zones. An air blower was used to supply oxygen to the microorganisms of aerobic zone. A peristaltic pump was employed to automatically furnish the system from the feed tank. The mixed liquor passing through the aerobic zones was recycled to the anoxic zone, and the sludge in the settling zone was returned back to the anaerobic zone. The reflux ratios of the mixed liquor and sludge were same, and set to 1. The sludge from a sewage treatment plant in Guangzhou was cultivated in a laboratory scale AAO treatment system with synthetic wastewater as feed. The synthetic wastewater with five different concentrations of DMP (>99% purity, Sinopharm Chemical Reagent Co., Ltd), which included 30, 40, 50, 60, and 80 μg L^−1^, was used.

In order to maintaining at a constant temperature of 25 °C, the work environment reactor system was controlled by the temperature control system. Dissolved oxygen (DO) was measured by the online dissolved oxygen meter (D53, HACH), and the concentrations of DO in anaerobic, anoxic and aerobic zones were within the scope of 0 to 0.30 mg L^−1^, 0 to 0.60 mg L^−1^ and 2.54 to 5.72 mg L^−1^, respectively. The mixed liquor suspended solid (MLSS) concentration of about 3000 mg L^−1^ was controlled in the reactor system. On the basis of changing the influent pump flow, hydraulic retention time (HRT) would be adjusted. Just as well sludge retention time (SRT) would be adjusted through altering the amount of the discharged excess sludge in the bottom of the settling zone. The continuous period of the operated system was one year. The basic information of Reactor system can been shown on supplementary information.

Gas Chromatography (Agilent 7890A, USA) and Mass Spectrometry (Agilent 5975, USA) (GSMS) was used to for determination and identification of the concentration of DMP. The detailed detection method was described by Huang *et al*.^32^. Mixed liquor suspended solid (MLSS) was measured according to Standard Methods^33^.

### Fuzzy wavelet neural networks (FWNN)

#### Structure of the proposed-FWNN

**Figure 2:**
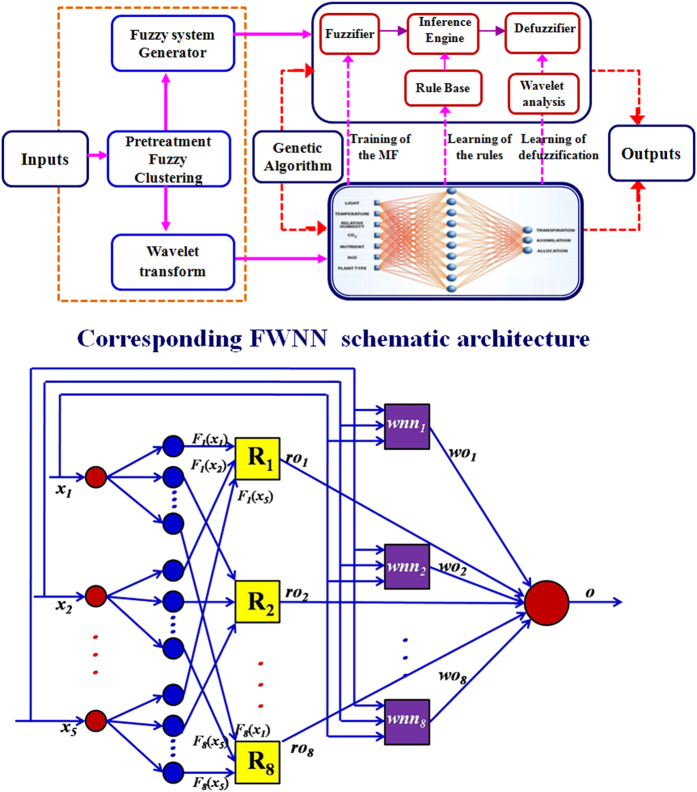
Architecture of the proposed fuzzy wavelet neural network system.

Figure 2 shows the structure of FWNN possessing five layers, which utilize wavelet functions as the neuron’s activation functions and realize fuzzy logical rules through five-layer NN^34^.

The first layer is input layer consisting of a group of processing units which are responsible for acceptance of data *x*_*1*_*; x*_*2*_*; … ; x*_*n*_ imported to the network. In this work, the number of input nodes is 5.

The second layer is the fuzzified layer. In this layer, the input characteristic variables from the first layer are translated into fuzzy variables through using the membership function, which is based on Gaussian function. The outputs of the layer are shown as below:


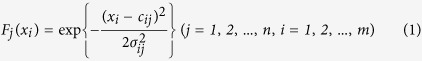


where *i* is the number of input singles and *j* is the number of the fuzzy rules in third layer. *c*_*ij*_ and *σ*_*ij*_ are the center position and the spread of Gaussian function. *F*_*j*_ (*x*_*i*_) is the membership function of the *i*th input variable with the *j*th fuzzy rule.

The third layer is called as fuzzy rule layer, which is used to realize the logical inference based on the fuzzy rule. Multiplication is used as AND operator here. The output of the *j*th node in this layer is


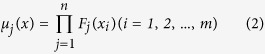


where *μ*_*j*_ (*x*) is the input single for the next layer and *n* is the number of fuzzy rule.

The fourth layer called as the wavelet network layer, which is used for data denoising transform. The product of the output from the layer 3 and 4 is set the input only to the layer 5. The output of the *j*th wavelet neuron in this layer is calculated by the following equation.


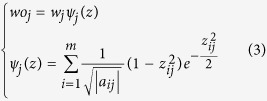


where 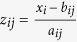 and *w*_*j*_ is the layer weight between *j*th wavelon and output node, the dilation (scale) parameter *a*_*ij*_ controls the spread of the wavelet and translation (shift) parameter *b*_*ij*_ determines its central position.

The fifth layer is the output layer. this layer calculates the overall output as the summation of output of previous layers. In this work, the output is the predicted effluent DMP of the FWNN model.

#### Training algorithm to optimize the proposed-FWNN

In this work, a hybrid learning algorithm based on gradient descent algorithm (GDA) and genetic algorithm (GA) was employed for adjust the parameters of the proposed FWNN, which included the center and width parameters of Gaussian functions (*c*_*ij*_ and *σ*_*ij*_), dilation and translation parameters of wavelet functions (*a*_*ij*_ and *b*_*ij*_), and the weight of the wavelet networks (*w*_*j*_)^35^.

GA was firstly used for the initialization of the proposed FWNN, then GDA was employed to obtain the optimal parameters of the FWNN. The advantages of the hybrid learning algorithm are obvious. Firstly, compared with only one optimization algorithm (GA or GDA), it brings more stable training process. Secondly, due to the “similarity” phenomenon existing in the population genetic of GA, GA with GDA can speed the convergence of the training process.

## Results and Discussion

### Kinetics of DMP degradation

In order to describing exactly the degradation behavior of DMP in AAO treatment system, the degradation models including biodegradation and sorption according to the Activated Sludge Model (ASM_2_) was developed based on the fate of DMP, which had been described by Huang *et al*.^32^,^36^


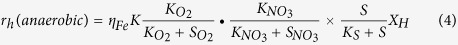



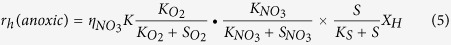



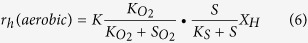


where *r*_*h*_ (*anaereobic), r*_*h*_ (*anoxic), and r*_*h*_ (*aerobic*) are the anaerobic hydrolysis process rate, anoxic hydrolysis process rate and aerobic hydrolysis process rate, respectively; *η*_*Fe*_ and 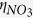 are anaerobic hydrolysis reduction factor and anoxic hydrolysis reduction factor; *K,* 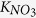
*and* 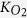 are saturation/inhibition coefficient for nitrate and saturation/inhibition coefficient for oxygen, respectively; 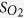
*and S*_*s*_ are dissolved oxygen and biodegradable substrate; *X*_*H*_ is heterotrophic biomass.

In addition, due to the uniformity of the mixed liquors in each reactor, the metabolic rate of DMP by microorganism in AAO system is uniform. Hence, the degradation rate of DMP in AAO treatment process could be described as the following equation:


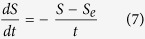


From what had been mentioned above, the model for describing the degradation rate of DMP could be simplified:


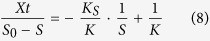


where 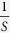, 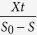, 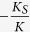 and 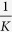 were represent as the variables of x, y, k and a, respectively. Therefore, equation (8) could be derived to a linear formula for relating the transformed values


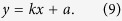


Base on the kinetic model of DMP degradation, kinetic parameters of DMP for the anaerobic degradation, anoxic degradation and aerobic degradation shown as in Tables S1–S3 (Supplementary Information) were determined. Thus according to the linear formula (Equation 9), the kinetic parameters of the models (K, Ks and η) shown in Table 1 were calculated. From Table 1, it can be seen that, the parameter η of DMP for the anaerobic degradation, anoxic degradation and aerobic degradation were 0.68, 0.80 and 1.00, respectively. That is because the mixed liquor passing through the aerobic zones was recycled to the anoxic zone, and the sludge in the settling zone was returned back to the anaerobic zone, which caused anaerobic sludge, anoxic sludge and aerobic sludge to have the similar characteristic. Thus. the removal efficiency of DMP in AAO treatment process was higher.

In order to assess the performance of the proposed models, the models were utilize for forecasting the DMP removal efficiency in AAO treatment process. It is very clear from Table 1 that the modeling approach gave good predictions. The forecasting errors were very small, mean absolute percentage error (MAPE) and root mean squared error (RMSE) of the model were both small, and the average value of relative errors were below 15%. The results clearly indicated the proposed model can describe exactly the degradation behavior of DMP in AAO treatment system due to the degradation model including biodegradation and sorption.

However due to the highly nonlinearity and complexity of degradation mechanism for DMP, traditional mathematical methods are hard to exactly to model and simulate the biodegradation process. Moreover, it was very difficult to establish the kinetics parameters of the mechanism model, artificial intelligence technique which can overcome the restrictions of the traditional modeling methods and efficiently approximate any nonlinear processes was used to model biodegradation of DMP. Therefore, With the self learning and memory abilities of NN, handling uncertainty capacity of FL, analyzing local details superiority of WT and global search of GA, a novel FWNN combines WNN with a TSK fuzzy model in order to enhance the function approximation accuracy.

### Modeling with FWNN

#### Data collection and preprocessing

The main objective of the data preprocessing is to determine suitable locations for the data required for modeling activities. In this work, the relationship between degradation of DMP and ORP, DO, pH, MLSS were selected to explore. Thus in order to develop the FWNN model, 50 sets of data was obtained from an AAO process 50 sets of data were obtained in the whole process, and 35 sets of measured data were selected as training samples and 15 sets of measured data were tested as forecast samples. In order to improve the performance of the model, normalization is one of the mostly used methods in data preprocessing In order to use the data into the network model for training, scaling was performed.

#### Development of the FWNN model

In this work, the FWNN model was used for forecasting the concentration of DMP. Through analyzing the mechanism of DMP in WWTP, the structure of FWNN model was determined, as shown in Fig. 2. After the initial structure and parameters of FWNN model were determined, a hybrid learning algorithm integrating improved genetic optimization and gradient descent algorithm was employed to train the network. After the structure and parameters of FWNN were optimized by GA, GDA was employed to update the parameters of the network.

#### Simulation results and analysis

**Figure 3:**
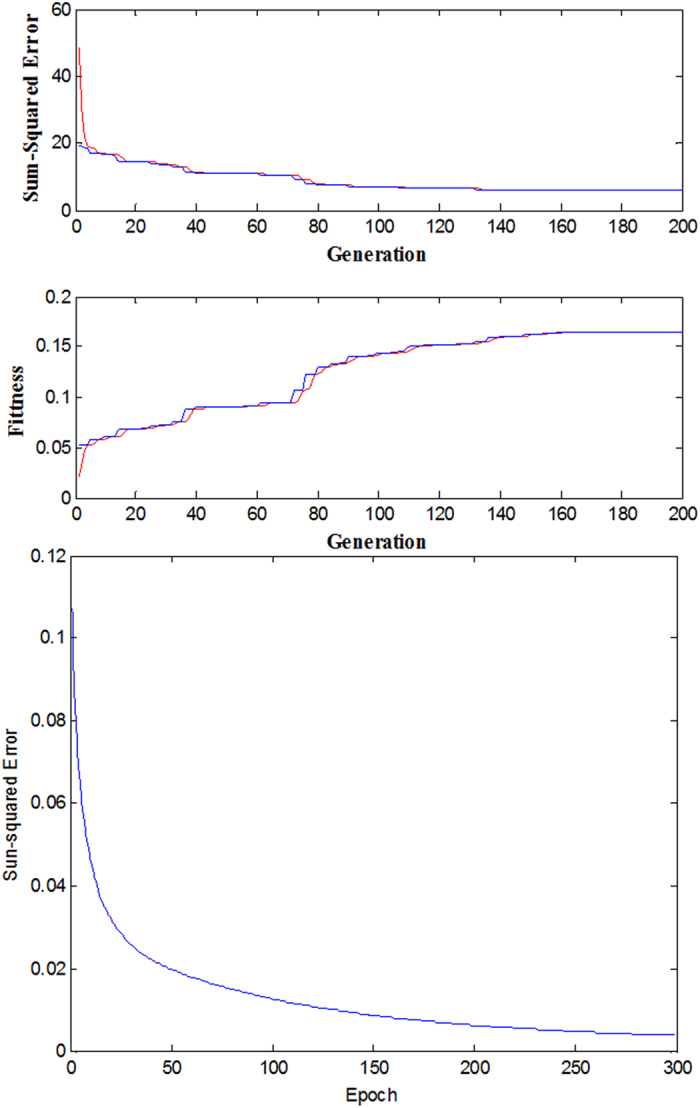
Training performance of FWNN based on hybrid GA-GDA algorithms.

In this work, the forecasting model based on FWNN was implemented on MATLAB. The initial population size N pop is 100, crossover rat Pc is 0.3, the interval of mutation Pm is 0.09, the maximum generation number was 200. Figure 3 shows the training process FWNN. From Fig. 3, it can be seen that the hybrid algorithm had rapid convergence ability and it met the target error rapidly. Thus, the center and width parameters of membership functions (*c*_*ij*_ and *σ*_*ij*_), dilation and translation parameters of wavelet functions (*a*_*ij*_ and *b*_*ij*_), and the weight of the wavelet networks (*w*_*j*_) were drawn, as shown in Tables 2 and 3.

**Figure 4:**
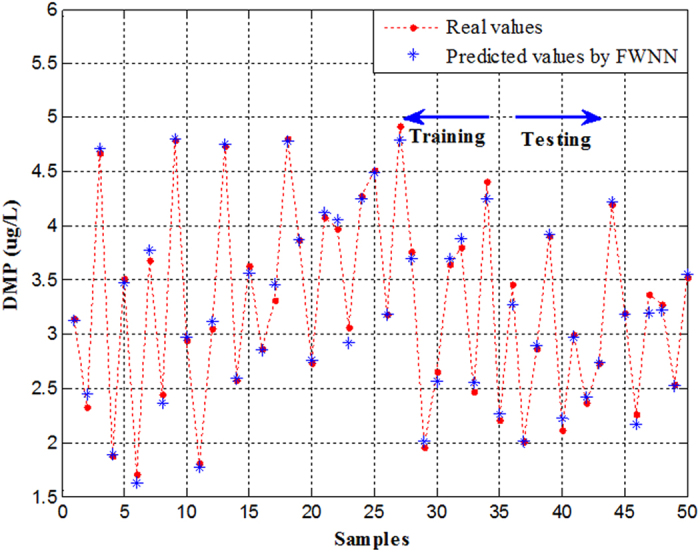
Compared actual output with predicted values based on FWNN.

**Figure 5:**
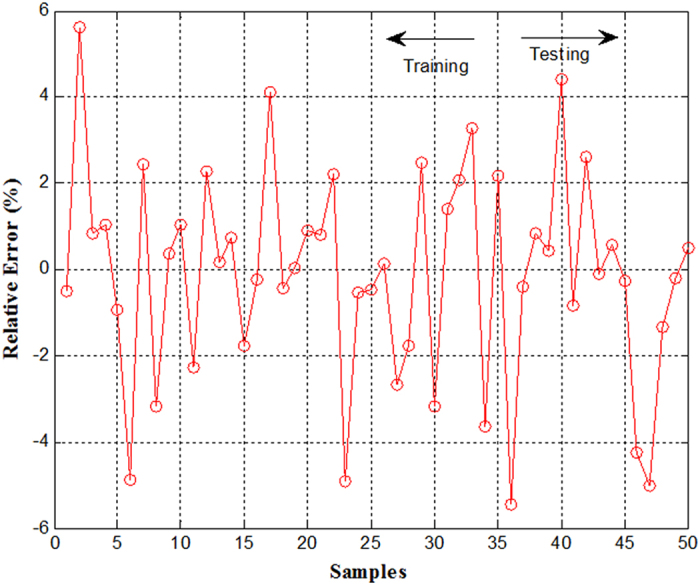
Error curve of training and testing in FWNN model.

The forecasting result of the proposed FWNN for testing datasets are demonstrated in Fig. 4. From Figs 4 and 5, it can be seen that the predicted values agree well with the observed values. On the basis of the simulation results of FWNN, the performance indexes of the proposed FWNN model for testing datasets are shown in Table 4. From Table 4, it can be seen that the proposed FWNN model achieved a very satisfactory prediction performance of effluent DMP in the AAO wastewater process. According to the high R^2^ of 0.9851, this case illustrates that the correlation between the predicted values and the observed values was excellent. Moreover, on the basis of the high R^2^, the FWNN model can explain 98.51% of the total variations. Furthermore, according to the values of the other descriptive performance indexes, which were are all nearly zero, it also revealed that the developed FWNN model showed a superior prediction performance, and there was only a small deviation produced by the developed FWNN model.

### Comparison of three models (FWNN, NN, and kinetic model)

In addition, in order to demonstrate the superiority of FWNN model, the developed FWNN model was compared with NN and kinetic model, and it can been seen that FWNN model has smaller RMSE (or MSE), MAPE and higher R^2^ values, as shown Table 4. When predicting, R^2^, MAPE, RMSE and MSE values were 0.9851, 1.8158%, 0.080 and 0.0064 using FWNN, respectively. However when using NN model with GA (GA-NN) and kinetic model, R^2^ were 0.9361 and 0.9036 respectively, MAPE were 5.0182% and 8.1017% respectively, and RMSE were 0.1658 and 0.2771, and MSE values were 0.02750 and 0.0768 respectively.

It is very clear from Table 4, FWNN model achieves better performances than GA-NN and kinetic model, which illustrates the FWNN model predicting the effluent DMP more accurate than GA-NN model and mechanism model. The results clearly indicates that the FWNN model had the high ability for extracting the dynamic behavior and complex interrelationships from various operation variables in the AAO wastewater treatment process.

Furthermore, compared with the kinetic model and NN model, the proposed FWNN has the advantages as follows: 1) FWNN, which makes effective use of self learning and memory abilities of NN, handling uncertainty capacity of FL and analyzing local details superiority of WT, could be constructed to enhance the abilities of approximation accuracy, convergence rate and generalization; 2) FWNN includes there search of the optimal definitions of parts of fuzzy rules, the determination of the sufficient number of layers and nodes, the parameter initialization of the structure and the training law; 3) Due to the good time-frequency localization characteristics of wavelets, FWNN possess better capability of learning and memory, and are superior to the convergence rate and accuracy; 4) FWNN has very important realistic meanings for optimizing operation parameters of reactor system, simulating the reactor system and enhancing the reactor system stability and efficiency.

## Conclusions

A novel FWNN for modeling and simulating biodegradation process of DMP was established in an AAO wastewater treatment process on the basis of the mechanism model. With the self learning and memory abilities of NN, handling uncertainty capacity of FL, analyzing local details superiority of WT and global search of GA, the reasonable forecasting performances had been achieved. Compared with NN model and kinetic model, FWNN model has smaller RMSE (or MSE), MAPE and higher R^2^ values and FWNN model achieves better performances. Therefore, FWNN is an efficient approach for modeling biodegradation process of DMP.

## Additional Information

**How to cite this article:** Huang, M. *et al*. A New Efficient Hybrid Intelligent Model for Biodegradation Process of DMP with Fuzzy Wavelet Neural Networks. *Sci. Rep.* **7**, 41239; doi: 10.1038/srep41239 (2017).

**Publisher's note:** Springer Nature remains neutral with regard to jurisdictional claims in published maps and institutional affiliations.

## Supplementary Material

Supplementary Information

## Figures and Tables

**Table 1:** Remove kinetics parameters of biodegradation of DMP.

Parameters	Fitting Eq. (*y* = *K*_*n*_ + *a*)	*R*^*2*^	*Ks*	*K*	*η*
Anaerobic reaction	y = 15.320x + 0.1033	0.9884	148.31	9.68	0.68
Anoxic reaction	y = 15.911x + 0.0880	0.9812	180.81	11.36	0.80
Aerobic reaction	y = 6.720x + 0.0701	0.9973	95.89	14.27	1

**Table 2:** Gaussian function parameters of FWNN.

Rules	pH		DMP_in_		DO		ORP		MLSS	
	*c*	*σ*	*c*	*σ*	*c*	*σ*	*c*	*σ*	*c*	*σ*
1	−0.5389	0.8539	−0.7172	1.8252	0.3490	2.0263	−0.2469	2.3372	−0.5437	10.4541
2	−0.7848	1.1428	−1.8480	−0.2853	−0.7183	3.2959	−1.7457	4.1071	−0.6529	4.9547
3	−0.5258	1.1863	0.8699	1.1137	0.6840	1.6768	0.5744	−1.7230	1.0631	−3.3109
4	−1.5954	−4.0123	0.9924	1.0084	1.3793	−11.0373	3.2149	−5.0486	0.0144	6.8924
5	0.3623	1.2555	−0.0754	0.5307	0.5229	1.2107	1.7393	123.1388	−0.0959	1.5124
6	−0.0739	2.4957	−0.0404	0.5781	−0.5956	3.3470	−0.1936	−3.1294	−0.3236	2.1482
7	0.3164	1.6734	−1.3487	1.3379	2.4650	3.8458	3.3962	6.7531	−0.0233	1.5783
8	0.4617	0.6413	−2.3481	26.4292	0.0942	2.6414	1.1412	291.9978	0.2500	1.4563

**Table 3:** The wavelet layer parameters of FWNN.

Rules	*w*	pH		DMP_in_		DO		ORP		MLSS	
		*a*	*b*	*a*	*b*	*a*	*b*	*a*	*b*	*a*	*b*
1	0.3637	0.1645	2.3704	2.1958	2.5965	−0.1480	3.7188	−1.8523	−8.5249	37.3451	479.7433
2	−1.6482	1.8295	2.5098	0.4551	−4.5770	1.8514	−8.3325	3.2568	2.1585	1.4103	7.6682
3	1.4141	5.3467	4.6311	−2.9073	−37.5183	−1.0283	−5.8732	−0.0282	−3.8119	−1.2960	−22.6885
4	2.3637	1.4598	15.2528	3.0360	0.2599	3.2318	−0.2844	−3.4982	−2.7673	−0.4079	8.6046
5	0.1636	1.6957	2.6433	1.4816	1.3972	−3.0100	−6.3009	1.9316	4.2983	−0.4130	−2.8903
6	2.3659	−0.0362	−2.1006	5.5159	8.0701	5.5763	4.7896	1.9583	−11.4900	1.8806	−9.9694
7	−4.0472	1.7613	25.9841	4.9113	3.0967	−0.6543	5.1786	3.9411	0.3220	−1.5826	−25.5946
8	1.1610	−0.2095	−8.3017	−0.4166	−8.1186	0.2922	−2.3139	−3.1274	−43.2687	0.2126	−2.5354

**Table 4:** Predicting performance using FWNN, NN and Kinetic model.

Item	FWNN Model	GA-NN Model	Kinetic Model
R^2^	0.9851	0.9361	0.9036
MAPE	1.8158	5.0182	8.1017
RMSE	0.080	0.1658	0.2771
MSE	0.0064	0.0275	0.0768
