# Supplementary material for: A New Efficient Hybrid Intelligent Model for Biodegradation Process of DMP with Fuzzy Wavelet Neural Networks
Source: Sci Rep. 2017 Jan 25;7:41239. doi: 10.1038/srep41239 (PMC5264161; doi:10.1038/srep41239)
Supplement: Supplementary Information [file srep41239-s1.doc]

**Supplementary Information**

**A New Efficient Hybrid Intelligent Model for Biodegradation Process of DMP with** **Fuzzy Wavelet Neural Networks**

**Mingzhi Huang^1*^, Tao Zhang^2^, Jujun Ruan^2^, Xiaohong Chen^1*^**

*^1^*Department of Water Resources and Environment, Guangdong Provincial Key Laboratory of Urbanization and Geo-simulation, Sun Yat-sen University, Guangzhou 510275, PR China

*^2^*School of Environmental Science and Engineering, Guangdong Provincial Key Laboratory of Environmental Pollution Control and Remediation Technology, Sun Yat-Sen University, Guangzhou 510275, PR China

*Corresponding author.

Address: Department of Water Resources and Environment, Sun Yat-sen University, Guangzhou, 510275, China Tel: +86-020-84114575, Fax: +86-020-84114575, E-mail: [huangmzh6@mail.sysu.edu.cn](mailto:huangmzh6@mail.sysu.edu.cn) (MZH), and eescxh@mail.sysu.edu.cn(XHC).

**The basic information of Treatment plant**

As shown in Fig. 1, the AAO treatment system made of polyethylene includes mainly four parts: one anaerobic zone with volume of 40 litres, one anoxic zone with volume of 40 litres, three aerobic zone with 160 litres and one settling zone. there were two motor-driven stirrers employed in anaerobic and anoxic zones. An air blower was used to supply oxygen to the microorganisms of aerobic zone. A peristaltic pump was employed to automatically furnish the system from the feed tank. The mixed liquor passing through the aerobic zones was recycled to the anoxic zone, and the sludge in the settling zone was returned back to the anaerobic zone. The reflux ratios of the mixed liquor and sludge were same, and set to 1.

The sludge from a sewage treatment plant in Guangzhou was cultivated in a laboratory scale AAO treatment system with synthetic wastewater as feed. The synthetic wastewater with five different concentrations of DMP, which included 30, 40, 50, 60, and 80 μg L-1, was used. Chemical organic demand (COD) was supplied from glucose. Ammounium nitrate (NH_4_NO_3_) and potasium dihydrogen phosphate (KH_2_PO4) were added to maintain the nitrogen and phosphorous sources in the system. The ratio of COD:N:P was kept at 100:7:1.

In order to maintaining at a constant temperature of 25℃,the work environment reactor system was controlled by the temperature control system. Dissolved oxygen (DO) was measured by the online dissolved oxygen meter (D53, HACH), and the concentrations of DO in anaerobic, anoxic and aerobic zones were within the scope of 0 to 0.30 mg L^-1^, 0 to 0.60 mg L^-1^ and 2.54 to 5.72 mg L^-1^, respectively. The mixed liquor suspended solid (MLSS) concentration of about 3000 mg L^-1^ was controlled in the reactor system. On the basis of changing the influent pump flow, hydraulic retention time (HRT) would be adjusted. Just as well sludge retention time (SRT) would be adjusted through altering the amount of the discharged excess sludge in the bottom of the settling zone. The continuous period of the operated system was one year.

**S1 Table. Computational sheet of anaerobic degradation kinetic parameters of DMP**

| T(h) | C_W_(ug/L) | C_S_(ug/gVSS) | S(ug/L) | 1/S | Xdt/dS |
| --- | --- | --- | --- | --- | --- |
| 0 | 11.890 | 22.473 | 59.983 |  |  |
| 1 | 10.660 | 20.493 | 54.516 | 0.018 | 0.391 |
| 2 | 9.620 | 18.587 | 49.395 | 0.020 | 0.418 |
| 3 | 8.787 | 16.687 | 44.496 | 0.022 | 0.437 |
| 4 | 7.960 | 14.990 | 40.039 | 0.025 | 0.480 |
| 5 | 7.170 | 13.457 | 35.967 | 0.028 | 0.526 |
| 6 | 6.857 | 11.897 | 32.316 | 0.031 | 0.586 |

**S2 Table. Computational sheet of anoxic degradation kinetic parameters of DMP**

| T(h) | C_W_(ug/L) | C_S_(ug/gVSS) | S(ug/L) | 1/S | Xdt/dS |
| --- | --- | --- | --- | --- | --- |
| 0 | 7.228 | 20.554 | 51.215 |  |  |
| 1 | 6.535 | 18.606 | 46.352 | 0.022 | 0.440 |
| 2 | 5.838 | 16.744 | 41.669 | 0.024 | 0.457 |
| 3 | 5.283 | 15.062 | 37.516 | 0.027 | 0.515 |
| 4 | 4.726 | 13.565 | 33.756 | 0.030 | 0.569 |
| 5 | 4.172 | 12.148 | 30.169 | 0.033 | 0.597 |
| 6 | 3.754 | 10.886 | 27.051 | 0.037 | 0.686 |

**S3 Table. Computational sheet of aerobic degradation kinetic parameters of DMP**

| T(h) | C_W_(ug/L) | C_S_(ug/gVSS) | S(ug/L) | 1/S | Xdt/dS |
| --- | --- | --- | --- | --- | --- |
| 0 | 4.341 | 17.649 | 42.111 |  |  |
| 1 | 3.257 | 14.396 | 34.065 | 0.029 | 0.266 |
| 2 | 2.872 | 11.383 | 27.232 | 0.037 | 0.313 |
| 3 | 2.106 | 9.195 | 21.784 | 0.046 | 0.393 |
| 4 | 1.783 | 7.131 | 17.044 | 0.059 | 0.452 |
| 5 | 1.414 | 5.573 | 13.340 | 0.075 | 0.578 |
| 6 | 0.782 | 4.476 | 10.362 | 0.097 | 0.719 |
